# 3D virtual histology of murine kidneys –high resolution visualization of pathological alterations by micro computed tomography

**DOI:** 10.1038/s41598-018-19773-5

**Published:** 2018-01-23

**Authors:** Jeannine Missbach-Guentner, Diana Pinkert-Leetsch, Christian Dullin, Roser Ufartes, Daniel Hornung, Bjoern Tampe, Michael Zeisberg, Frauke Alves

**Affiliations:** 1 Institute of Diagnostic and Interventional Radiology, University Medical Center Goettingen, Robert Koch Str. 40, Goettingen, Lower Saxony 37075 Germany; 2 Clinic of Hematology and Medical Oncology, University Medical Center Goettingen, Robert Koch Str. 40, Goettingen, Lower Saxony 37075 Germany; 3 Italian Synchrotron Light Source “Elettra”, Trieste, 34149 Italy; 4 Translational Molecular Imaging, Max Planck Institute for Experimental Medicine, Hermann-Rein-Str. 3, Goettingen, Lower Saxony 37075 Germany; 5 Group of Biomedical Physics, Max Planck Institute for Dynamics and Self-Organisation, Am Fassberg 17, Goettingen, Lower Saxony 37075 Germany; 6 Department of Nephrology and Rheumatology, University Medical Center Goettingen, Robert Koch Str. 40, Goettingen, Lower Saxony 37075 Germany

## Abstract

The increasing number of patients with end stage chronic kidney disease not only calls for novel therapeutics but also for pioneering research using convincing preclinical disease models and innovative analytical techniques. The aim of this study was to introduce a virtual histology approach using micro computed tomography (µCT) for the entire murine kidney in order to close the gap between single slice planar histology and a 3D high resolution dataset. An *ex vivo* staining protocol based on phosphotungstic acid diffusion was adapted to enhance renal soft tissue x-ray attenuation. Subsequent CT scans allowed (i) the detection of the renal cortex, medulla and pelvis in greater detail, (ii) the analysis of morphological alterations, (iii) the quantification of the volume as well as the radio-opacity of these portions and (iv) the quantification of renal fibrotic remodeling based on altered radio-opacity using the *unilateral ureteral obstruction* model. Thus, virtual histology based on *PTA contrast enhanced CT* will in future help to refine the outcome of preclinical research on kidney associated murine disease models.

## Introduction

Kidney diseases, either caused by hypertension or diabetes mellitus or as a consequence of renal senescence, remain an increasing challenge of industrialized countries. Up to now, the most common method to assess renal disease models is still the labor intensive histological sectioning at the endpoint of the experiment, which only provides planar information about a small area. Moreover, due to paraffinization and sectioning, the samples are often destroyed. Therefore, novel research tools are needed to analyze anatomical alterations in the entire organ to understand pathomechanisms or to evaluate novel therapeutic strategies.

Preclinical CT approaches offer the non-invasive three-dimensional (3D) visualization of anatomical structures *in vivo* within seconds and in a reasonable resolution^1^. CT relies on the inherent contrast of dense tissue structures such as bone and teeth in comparison to the surrounding poor contrast of soft tissue. To depict soft tissue like muscle or peritoneal organs by CT, an enhancement of contrast is required. The application of iodine based contrast agents (CA) *in vivo* has for instance been used for the detailed depiction of blood vessels over time or for the delineation of the urinary tract making use of the renal excretion of the CA^2^. The combination of the advantages of high resolution planar 2D microscopy, characterized by a small field of view, with the benefits of 3D CT data sets of whole organs with high spatial resolution, would be beneficial to visualize morphological alterations in complex tissues.

An alternative diagnostic approach is the analysis of contrasted *ex vivo* specimens, e.g. murine hearts and embryos with experimental CT techniques^3^. To use the whole potential of such an *ex vivo* CT approach with sufficient soft tissue differentiation, the diffusion of CA through the tissue is required. A possible CA for this purpose is phosphotungstic acid (PTA), a compound of the histochemical Masson-Goldner-trichrome (MGT) staining for connective tissues^4^. Tungstic ions in particular are known to bind collagen and fibrin fibers^5,6^ and to compete as a “colorless acid dye” with other dyes like fuchsin for accessible amino groups in tissue proteins^7^. The compactness of the tissue constituents defines the permeability for PTA, in dependence of the density of the cells or the deposition of extracellular matrix (ECM)^8^. Tungsten, a transition metal, is an ideal contrast agent for x-ray techniques due to its high atomic number of 74. *Post mortem* PTA staining was already successfully applied for high resolution CT imaging of embryos and insects^9–11^, to spatially analyze murine brains and developing mouse hearts^12–14^ and to assess atherosclerotic plaques in a mice^3^.

The possibilities of high resolution CT imaging of kidneys are as diverse as kidney diseases themselves. Besides cystic kidney diseases, chronic kidney diseases (CKD) are common nephropathies, which are characterized by glomerulosclerosis, tubular atrophy and interstitial fibrosis^15^. CKD progression to end-stage renal disease can neither be cured nor stopped or reversed^16,17^. Nephropathies increasingly emerge as an urgent problem for clinicians because the susceptibility for kidney injury increases with age, particularly due to the frequent use of nephrotoxic medications like non-steroidal anti-inflammatory drugs, diuretics and aminoglycoside, and the increase of the older population in the western world^18^. Experimental mouse models are used, which simulate the complex nature of renal failure like chronic allograft nephropathy^19^ or CKD^20^, to determine the negative effects of novel therapeutic agents on kidney function, such as tubulointerstitial damage or drug-related glomerular injury^21^.

The aim of the study was to establish a feasible protocol for *ex vivo* staining of murine kidneys with PTA in order to achieve an enhanced contrast for CT examination without damaging the organ. We show that µCT data sets of PTA stained kidneys can be used for virtual histology in 3D, depicting detailed characteristic morphological structures as well as pathological features of renal fibrosis and renal cysts of various diameters. Moreover, we demonstrate that the extension of the fibrosis can be quantified and subsequently analyzed by histological and immunohistochemical (IHC) procedures.

## Results

### Micro-CT imaging of PTA stained kidneys allows detailed 3D virtual histology

**Figure 1:**
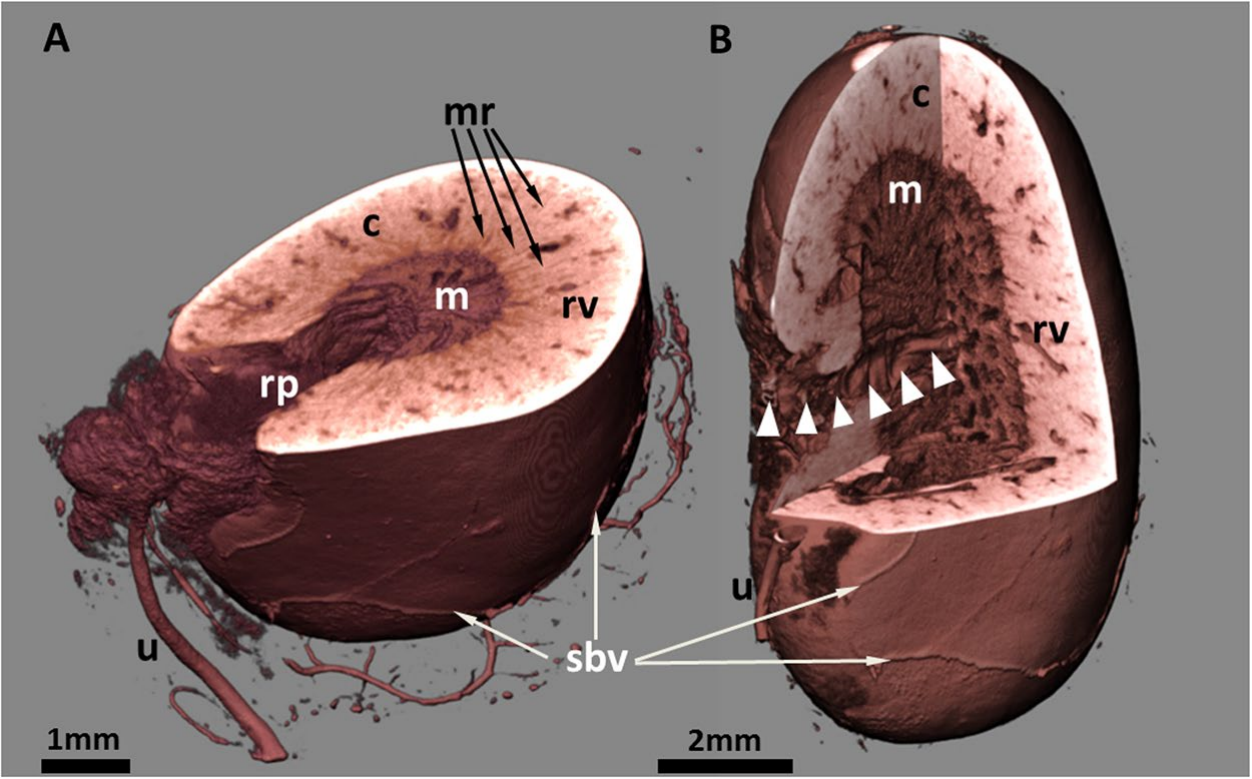
Virtual cut sections through the volume rendering presentation of a murine kidney. The PTA stained organ was scanned by µCT and displays morphological features at the surface like superficial blood vessels (sbv) and the ureter (u). Moreover, details of the inner anatomical structures are clearly distinguishable. (**A**) The dense cortical region (c) with prominent renal vessels (rv), and medullary rays (mr, arrows), passing the cortex, the medulla with parallel organized tubules and the renal pelvis (rp) with nearly no PTA contrast. (**B**) The orientation of the virtual cut allows tracking a big renal vessel from the hilus to the medulla (arrow heads) and offers insights into the 3D renal organization of cortex and medulla.

In order to generate a sufficient soft tissue contrast for CT visualization of renal morphology, explanted kidneys were stained for four to 18 days in PTA solution until complete penetration of PTA was achieved in the organ. To verify this, the kidneys were scanned at certain time points using the *QuantumFX* CT, achieving a voxel size of 40 µm. The criteria for a complete PTA penetration were: (i) a homogeneous contrast within the structural regions of the soft tissue without the occurrence of unstained areas, and (ii) a negative contrast only for physiologically developed cavities like the renal pelvis or blood vessels (Fig. 1). After verification of complete PTA staining by CT, the kidneys were individually embedded in agarose or paraffin filled vials (1.8 ml) to avoid movement artifacts and subsequently scanned with the *Phoenix nanotom* µCT, achieving a voxel size of 12.5 µm.

**Figure 2:**
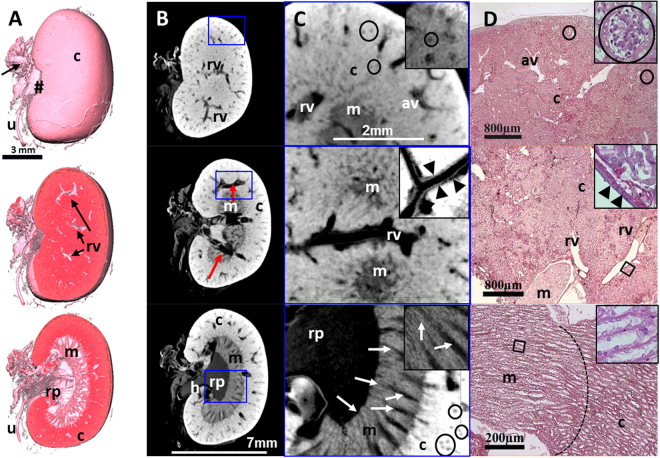
Virtual cut through representative 3D and 2D images of a µCT data set from a single PTA perfused murine kidney, stained for 13 days. (**A**) Upper panel: the volume rendering 3D image of the kidney illustrates the renal capsule (c), but also associated structures like the ureter (u), connective tissue at the sinus (#) and supporting blood vessels (arrows) can be depicted. Middle and Lower panel: a superficial frontal cut allows the visualization of the renal blood vessels (rv) within the renal cortex (c) whereas an image of a deeper layer reveals all characteristic anatomical structures of the kidney: the dense cortex with sections of small arcuate vessels (arrows), the looser tubular system of the renal medulla (m) and the collecting renal pelvis (rp). (**B**) 2D sections of the same kidney demonstrate different intensities of PTA staining within the renal structures. The blood vessels are not contrasted (red arrows). (**C**) Upper panel: a higher magnification shows even small furcation of the arcuate vessels (av) within the cortex. Small black dots (circled, at higher magnification in the small frame) correspond to glomeruli. Middle panel: renal vessels are almost devoid of contrast, although the vessel wall (arrowheads, at higher magnification in the small frame) can be clearly depicted. Lower panel: the higher magnification allows for depiction of the medullar tubular system with distinct tubules (arrows, at higher magnification in the small frame) discharging into the renal pelvis (rp). (**D**) Corresponding H&E stained histological sections confirm the renal organisation with its cortical structures, such as arcuate vessels and glomeruli (circled, at higher magnification in the small frame) a larger renal vessels (rv) and the pelvis (rp). Middle and Lower panel: in histological planar projections, renal vessels (arrow heads, at higher magnification in the small frame) and the intersection from glomerular organisation of the cortex (c) to the tubular system of the medulla (m, dashed line) are clearly distinguishable because of a changing orientation of the tubuli (at higher magnification in the small frame).

Due to the PTA binding to tissue proteins - depending on the charge and size of molecules and the diffusion time within the kidney - we were able to depict the renal capsule and, additionally, other structural parts of the kidney. In particular the renal cortex, the medulla with the renal pyramid and the renal pelvis as well as the discharging unit consisting of the hilus and the ureter were visualized in 3D in arbitrary angles (Fig. 1A). The gradual reduction of brightness we observed from the outer capsule to the inner medulla and pelvis (Fig. 1A,B) indicates that the amount of tissue and its compactness within the renal structure decreases from the outer cortex to the cavernous pelvis. Hollow structures such as medullary rays and collecting ducts with different diameters and supporting vessels (e.g. lobular/interlobular vessels) were not contrasted by PTA and appeared dark in the 2D CT data sets (Fig. 2A,B). Data sets at higher magnification even enabled the display of the endothelium of renal blood vessels, as well as tubules, single collecting ducts and glomerular corpuscles as small dark spots of approximately 50–100 µm (Fig. 2C).

Microscopic analysis of the corresponding hematoxilin & eosin (H&E) stained kidney paraffin sections confirmed the anatomical structures visualized by the CT/µCT data sets (Fig. 2D) and depicted the same structures as µCT on single cell level; for example, the microstructure of the kidney and the renal corpuscles, followed by the convoluted tubules to the collecting ducts.

### PTA perfusion followed by e*x vivo* PTA staining combines visualization of renal vascularization with morphological features

**Figure 3:**
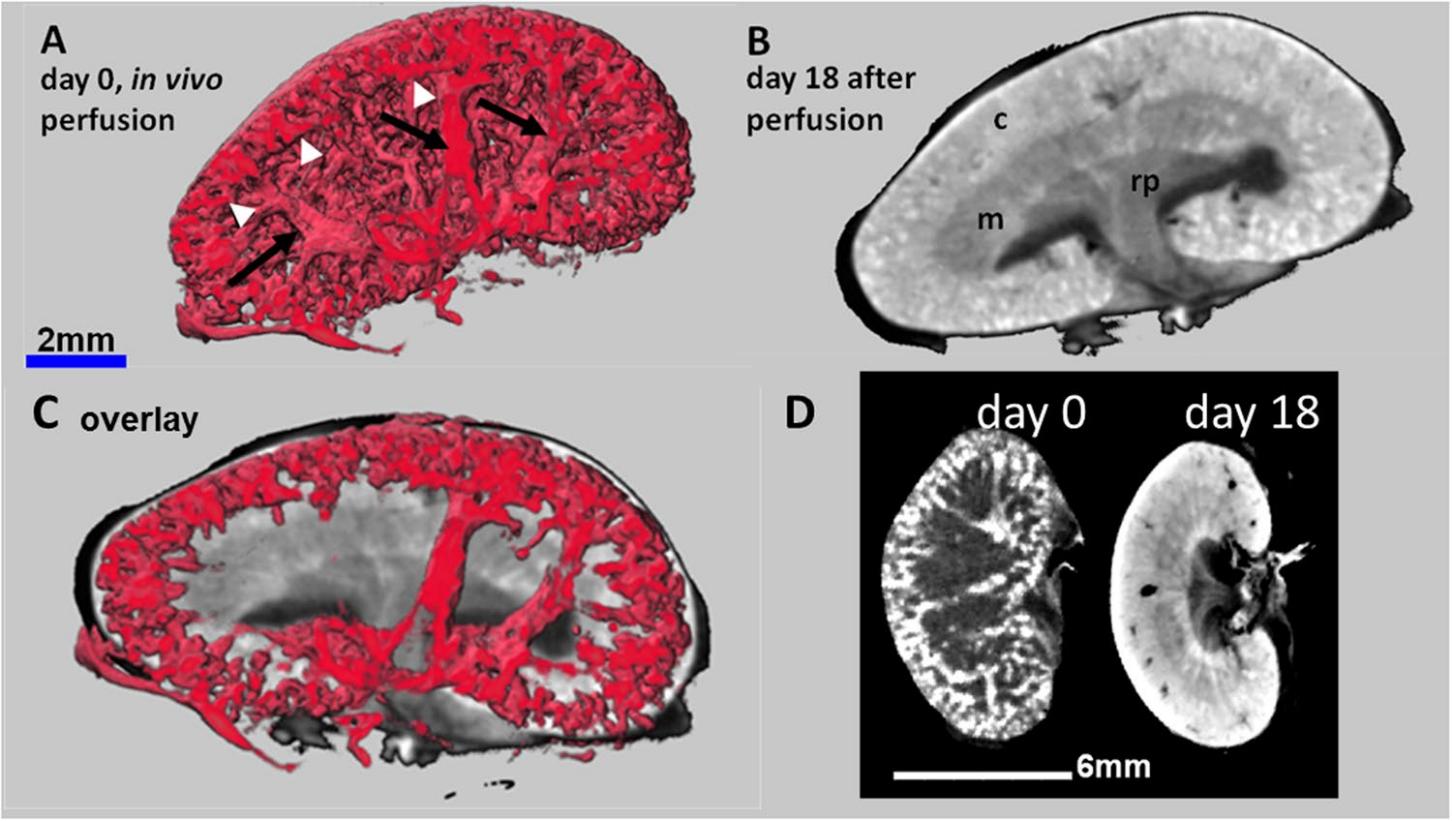
CT images of kidneys after *in vivo* PTA perfusion combined with *ex vivo* PTA staining. (**A**) Quantum CT scan of a kidney of a healthy mouse after *in vivo* PTA perfusion shows i) PTA filled blood vessels (arrows), bifurcated from the main renal vessels (not shown) to the arcuate vessels, and ii) the associated dense lattice of capillaries in the cortex in 3D (arrow heads). (**B**) In contrast, CT images of the same excised kidney after additional *ex vivo* PTA staining for 18 days visualizes the typical diffusion pattern, allowing the discrimination of anatomical structures such as cortex (c), medulla (m) and renal pelvis (rp). The vessel contrast is completely blurred. (**C**) An overlay of adjusted section planes of perfusion and diffusion data sets enables the co-localization of the blood vessels with the anatomical details of the kidneys. (**D**) 2D views of the same kidney at day 0 (kidney only perfused with PTA) and day 18 (kidney PTA perfused *in vivo* and PTA stained *ex vivo*) demonstrate the vessel contrast after PTA perfusion and the morphological contrast after PTA diffusion.

In order to increase the contrast of the supporting blood vessels in the kidney, mainly represented by the renal vein, the renal artery and their increasing number of branches towards the cortical periphery, we transcardially perfused mice with a 5% PTA solution before dissection and scanning by *QuantumFX* CT. Although the arteries and veins could not be clearly distinguished, a dense lattice of blood vessels from the segmental arteries and veins up to the small organisation grade of dense cortical arcuate vessels was depicted in detail *ex vivo* (Fig. 3A,D), approximately 20 min after PTA perfusion. To increase the morphological contrast, the kidneys were additionally immersed *ex vivo* in PTA solution according to the standard procedure for up to 18 days. While the long term diffusion of the PTA staining solution into the kidneys contrasted the renal cortex, medulla and renal pelvis, the perfused PTA solution within the vessels had completely leaked out (Fig. 3B,D). The composite image obtained by an overlay of the two 3D data sets with the software SCRY enabled the simultaneous depiction of the renal vessel lattices with their typical branching architecture and their underlying structural regions of renal cortex and medulla (Fig. 3C).

### PTA staining of kidneys does not lead to shrinkage of the tissue

To determine the degree of possible tissue shrinkage due to PTA staining, freshly isolated kidneys from five female healthy mice, eight to nine months of age, were scanned with the *QuantumFX* CT prior (native), and directly after PTA staining (twelve days). Before performing another CT scan, the samples were drained and embedded in paraffin, in order to mimic the preparation for histological procedures. The volumes of the native, the PTA stained and the paraffin embedded kidneys were assessed in the CT data sets following threshold based segmentation implemented in the software *SCRY*.

Volumes of PTA stained organs were on average (±standard deviation) 11.8% (±5.57%) larger than those of unstained kidneys, suggesting a slight swelling of the organs. Surprisingly, the following standard procedures such as dehydration and paraffin embedding led to an average shrinkage of the kidneys by 18.7% (±7.26%) in comparison to native kidneys. Volumes of unstained kidneys embedded in paraffin could not be depicted due to the insufficient contrast between paraffin and kidney in the CT data sets. In conclusion, processes of dehydration and paraffin embedding are responsible for a loss of volume of about one-fifth of the initial kidney volume, which should be taken into account when comparing CT scan results with histological findings.

### CT data sets enable the quantitative analysis of renal structures

**Figure 4:**
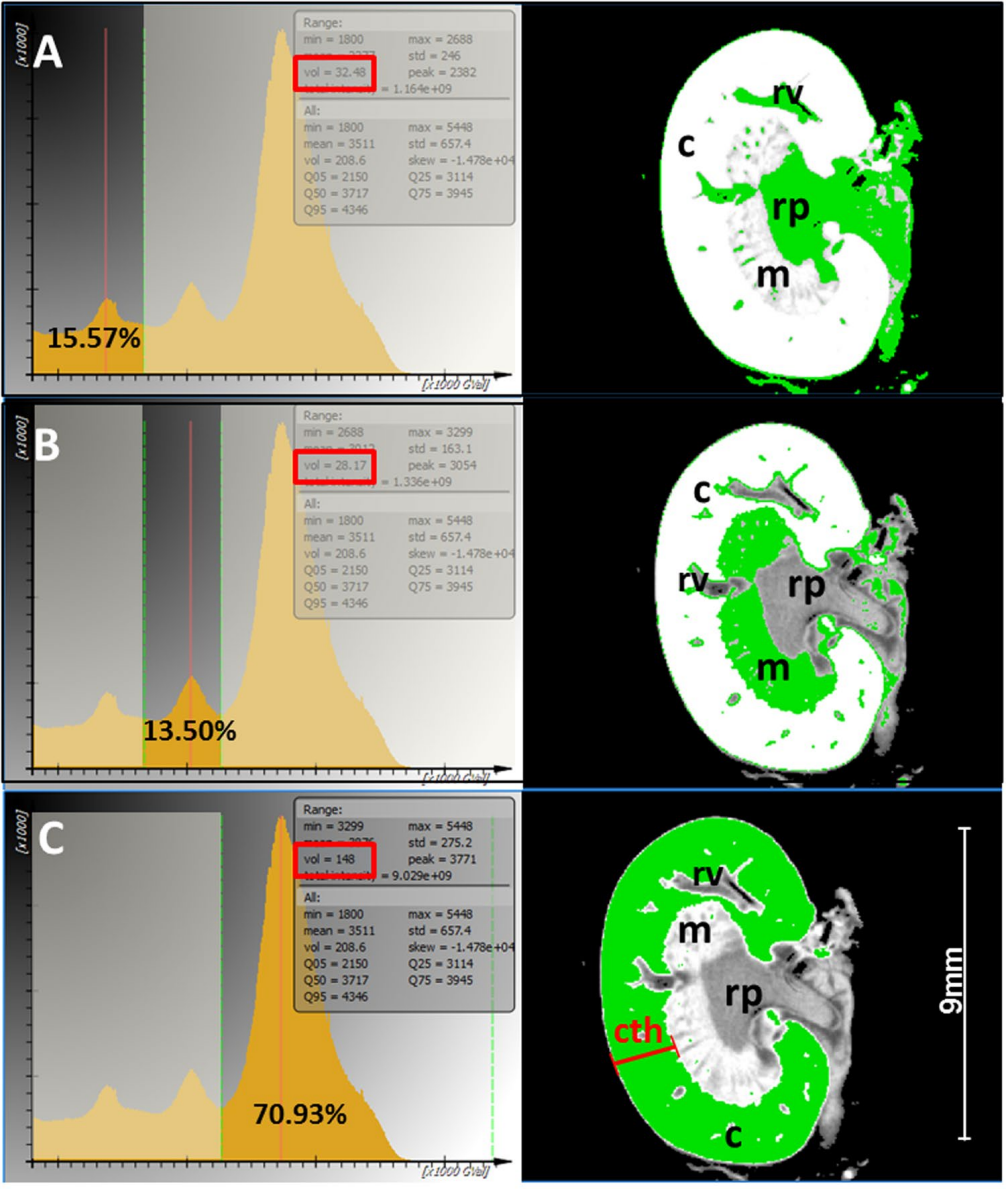
Semi-automatic and quantitative analysis of anatomical structures in the healthy kidney. Left panel: Representative radio-opacity histograms of the entire kidney of a four months old male mouse, after 13 days of PTA staining are shown and allow the volumetry of the renal pelvis, medulla and cortex due to the uptake of PTA according to tissue opacity. The different classes of grey values are represented as partially overlain Gaussian distributions. The depiction of inflection points allows the segmentation of the curve into different portions, reflecting pelvis and associated tissue (**A**) Medulla (**B**) and pelvis, which can be quantified absolutely (frames) or relatively as the percentage of the whole kidney volume. The distribution of the specific grey value portion is illustrated by 2D CT images of frontal sections of the corresponding kidney (right panel). (**A**) Left panel: 15.57%, which equates to 32.5 mm^3^ (red frame) of the whole kidney volume is represented by the left grey value portion to the first inflection point. Right panel: This portion of low radio-opacity belongs to parts of the nearly unstained renal pelvis (rp) and the lumen of renal vessels (rv), depicted in green within the 2D frontal section of the kidney. B, Left panel: A middle class of grey values of 13.5%, which equates to 28.2 mm^3^ (red frame), defined by two inflection points of the surrounding envelopes, correlates to the medulla (m) and to blood vessel walls within the cortex (c) as shown in the right panel, according to the distribution of the green color. (**C**) Left panel: The major volume portion (70.93%), which equates to 148 mm^3^ (red frame) of the kidney is represented by a Gaussian curve in the range of the highest radio-opacity. Right panel: This distribution correlates with the most compact structure within the kidney – the cortex (c), depicted in green. The cortex thickness (cth) is defined as distance from renal capsule to the border of the medulla (red line).

*Ex vivo* PTA staining of the kidneys not only allowed the qualitative visualization of 3D kidney morphology but enabled the exact quantification of certain renal regions such as cortex, medulla and pelvis, due to the distinct uptake of PTA in these functional structures. Using the software *SCRY*, a histogram was generated from the CT data of each isolated and PTA stained kidney, representing the different distribution of x-ray absorption or radio-opacity throughout the kidney, displayed by grey values (GV) within a defined volume of interest (Fig. 4). As the extent of PTA accumulation differs in various structures of the kidney, the PTA content defines the average x-ray absorption within the organ and thereby leads to a specific grey value distribution within a volume specific histogram (Fig. 4).

The histogram of each kidney consists of a curve, which represents the air within the scanned volume and of three overlapping Gaussian curves, corresponding to three GV classes, which represent the three structural sections: renal cortex, medulla, and pelvis. Furthermore, the histogram contains information about the volume of the scanned kidney. By identifying the inflection points of the envelope of the histogram, which define the borders between the grey scale classes, the specific volume within these inflection points can be defined and used for the quantification of all volume portions, analogous to the morphological structures (Fig. 4). This approach not only enables the measurement of the entire renal volume, but also the determination of the absolute and relative volume portions of pelvis (Fig. 4A), medulla (Fig. 4B) and cortex (Fig. 4C) as parameters for the quantification of structural alterations within the kidney, for example during kidney dysfunction.

Since the loss of renal cortical thickness is a surrogate marker for renal dysfunction and renal age, we analyzed the renal cortical thickness of eight kidneys from four healthy mice of different sex and age using CT based virtual cross sections. The high resolution CT data sets were oriented in a standard frontal view of the central section, allowing an accurate measurement from the renal capsule to the beginning of the medulla (Fig. 4C). Because of the strong contrast between the intensively stained renal cortex and the weaker stained medulla, a clear delineation of both structures allowed us to define the distance between renal capsule and the border of the renal medulla (Fig. 4C). In order to calculate the mean renal cortical thickness, distances from at least five different positions and the entire volume of each kidney were depicted as mean renal cortical thickness with 1.78 ± 0.12 mm. To exclude effects of the kidney size on the variation of the renal cortical thickness, the surface of the kidney was calculated to generate an index of the square root of the kidney surface divided by the mean of the renal cortical thickness (Table 1). We found a stable correlation between the square root of the kidney surface and the renal cortical thickness (Index = 3.445 ± 0.151), indicating that a smaller renal cortical thickness in these healthy mice is associated with a smaller size of the entire kidney. To estimate the whole relative volume of the renal cortex instead of the 2D thickness, the inflection point between the envelope for the medulla and cortex was determined within a volume specific histogram. The volume of the renal cortex is represented by the sum of the brightest voxel class from this inflection point to the maximum density value (Fig. 4B). The obtained relative volume fraction of the cortex of the collective of healthy mice ranged from 63.6% for the 19.5 months old female mouse to 80.8% for the 8 months old male mouse (mean: 72.0% ± 5.5%). Thus, the kidneys of the oldest mouse possessed the thinnest renal cortex and the smallest relative cortex volume, suggesting a correlation between loss of renal thickness/cortical volume and renal age (Table 1).

**Table 1:** Quantification of thickness and volume of murine kidneys, exemplified for murine kidneys from animals of different sex and age.

individual	V_kidney_	V_pelvis_	V_medulla_	V_cortex_	mean cortex thickness	A_kidney_ mm^2^	Index √A_kidney/_Cth
C57/Bl6, ♂, 8 month	316.7 mm^3^	8.70%	13.3%%	78.1%	2.147 mm	46.46 mm^2^	3.175
	341.1 mm^3^	7.53%	11.68%	80.79%	2.115 mm	48.82 mm^2^	3.304
C57/Bl6, ♂, 12 month	222.4 mm^3^	12.76%	12.88%	74.37%	1.730 mm	36.71 mm^2^	3.502
	195.2 mm^3^	13.40%	18.20%	68.40%	1.692 mm	33.65 mm^2^	3.428
C57/Bl6, ♀, 19.5 month	188.1 mm^3^	10.42%	20.22%	69.37%	1.595 mm	32.83 mm^2^	3.592
	201.2 mm^3^	11.56%	24.84%	63.62%	1.621 mm	34.34 mm^2^	3.615
C57/Bl6 ♂, 4 month	200.2 mm^3^	16.20%	13.30%	70.5%	1.724 mm	34.22 mm^2^	3.393
	195.2 mm^3^	15.57%	13.5%	70.93%	1.628 mm	33.63 mm^2^	3.55
mean	232.5 ± 60.67 mm^3^	12.02 ± 3.082%	15.99 ± 4.629%	72.01 ± 5.524%	1.781 ± 0.2213 mm	37.58 ± 6.340 mm^2^	3.445 ± 0.151

The volume of the whole kidney and the structural regions pelvis, medulla and cortex were analyzed, as well as the mean 2D cortical thickness and the surface (A) of the whole kidney. To exclude any effects of kidney size on the variation of the renal cortical thickness, an index was calculated: the square root of the kidney surface divided by the mean of the renal cortical thickness. The index indicates a strong correlation between cortical thickness and kidney size.

### Altered morphology of kidneys is depicted by CT after PTA-staining

**Figure 5:**
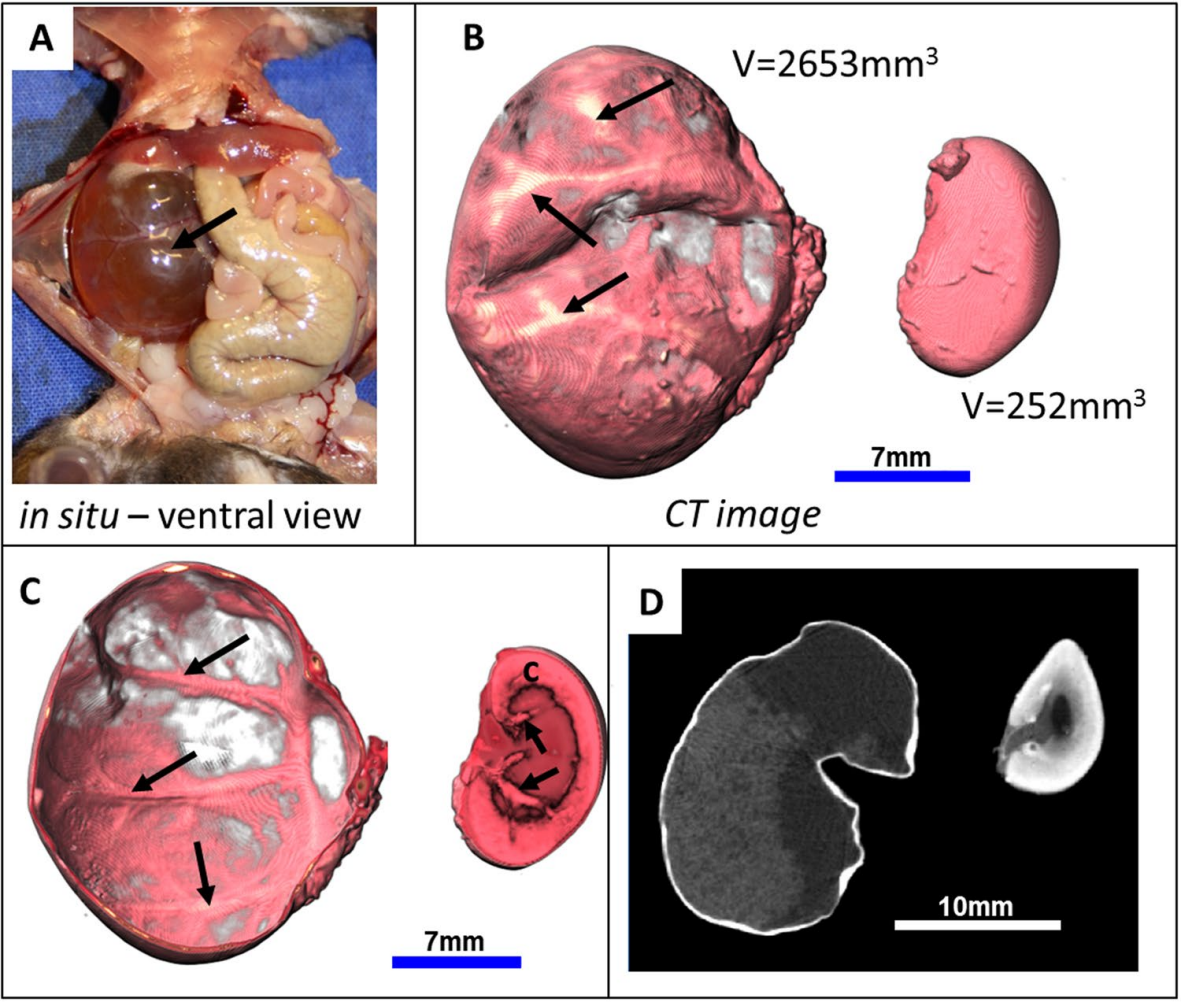
Visualization of a unilateral murine cystic kidney. (**A**) The *in situ* macroscopic view during dissection shows a degenerated right kidney that contains a large single cyst filled with urine. (**B**) Representative 3D volume rendering µCT images of isolated kidneys after 13 days of PTA staining allow a reliable volumetric measurement and accentuates the uneven surface and superficial blood vessels (arrows) of the cystic left kidney (left) in comparison to the normal sized contralateral kidney (right). (**C**) A frontal cut displays, besides the blood vessels (arrows), only the thin renal capsule in comparison to the left kidney with its contrasted renal cortex, containing thick branches of renal vessels (arrows). (**D**) 2D virtual sections of the kidneys demonstrate a diffuse enhancement in the cystic kidney, representing most probably a precipitate of PTA within the liquid (left) and show the normal distribution of PTA in the normal kidney, depicting cortex, medulla and renal pelvis (right).

In order to explore the PTA accumulation in murine dysplastic kidneys, we applied our CT based virtual approach to a PTA stained cystic and a cirrhotic kidney, which occurred spontaneously in two senescent mice. An enhanced peritoneal mass on the right side of a one year old mouse emerged as a unilateral, single cyst within the kidney after dissection (Fig. 4A). The enlarged right kidney was urine filled and the renal parenchyma was nearly completely degenerated and transparent. Both kidneys were PTA stained for thirteen days and scanned thereafter with the *QuantumFX* CT. While the unaffected kidney showed the regular distribution of PTA within the cortex, medulla and renal pelvis, the cystic kidney displayed contrast only within the thin renal capsule and the rudimentary renal parenchyma (Fig. 5B,C). This contrast was sufficient to determine the volume of both kidneys (Fig. 5B) by segmentation of the CT data set. The cystic kidney displayed a tenfold larger volume (2653 mm^3^) than the one of the contralateral normal kidney (251.6 mm^3^). The most enhanced contrast was observed in the peripheral blood vessels on the surface of the kidney (Fig. 5B,C). The 2D CT section of the cystic kidney demonstrated a diffuse, slight enhancement of contrast within the kidney, associated with the PTA that entered into the liquid that filled the kidney (Fig. 5D).

**Figure 6:**
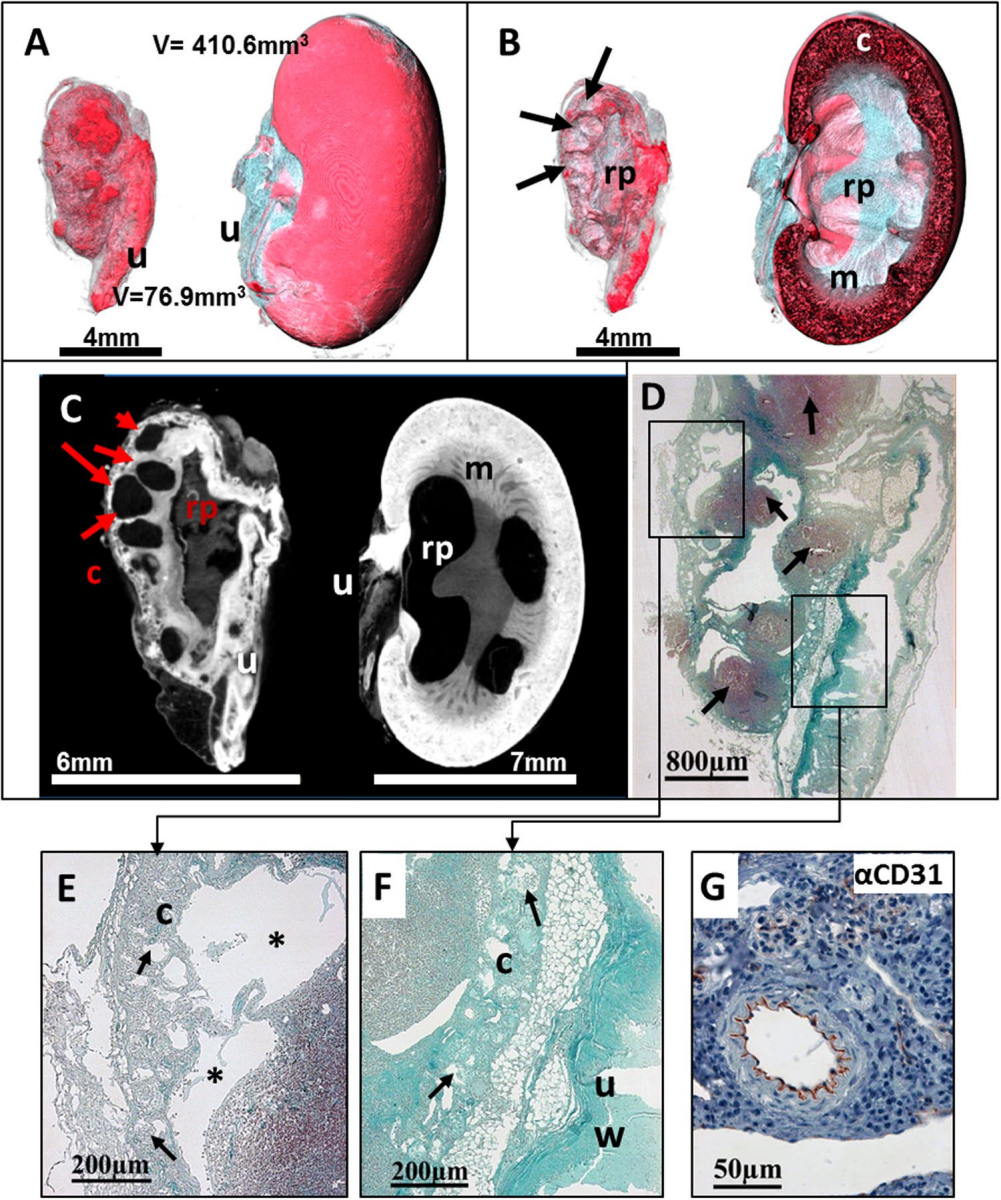
Visualization of a murine cirrhotic kidney. (**A**) Volume rendering µCT images display the cirrhotic kidney (left) and the slightly augmented kidney (right), with its associated ureter (u). In comparison to the smooth surface of the right kidney with a size of 410.6 mm^3^, the smaller left cirrhotic kidney with a volume of 76.9 mm^3^ appears with a blistered, irregular surface. (**B**) A virtual cross section of the CT image of the left kidney reveals multiple small cysts (black arrows) within the thinned parenchyma and a degenerated and cavernous renal pelvis (rp). Cross section of the right kidney reveals the stratification of cortex (c) medulla (m) and renal pelvis (rp), although the widened pelvis and the reduced medullar contrast point to a beginning pathological alteration. (**C**) 2D sections reveal multiple cysts of different sizes (red arrows) within the irregular shaped cortex (c) and depict structures with a notable contrast representing the renal pelvis (rp) as well as the ureter (u) within the cirrhotic kidney. In comparison to the cirrhotic kidney, the right kidney shows a stronger PTA staining within its cortex whereas only a slight contrast can be seen in the wall of the ureter (u). (**D**) Corresponding histological sections of the cirrhotic kidney, stained with Masson-Goldner trichrom (MGT), confirm fibrotic and inflammatory remodeling within the kidney as obtained by µCT image (**B**–**E**) At higher magnification, the left photomicrograph shows the thinned parenchyma without distinct differentiation between cortex and medulla (Left upper frame) with an irregular central renal pelvis (Right lower frame). Note that inflammatory cells (arrows) accumulate in the cortex as a sign of inflammation. Higher magnification of the marked frames shows spacious cystic areas (*) within the thin cortex (c) and cystic degenerated glomeruli (arrows, middle panel). The wall of the ureter (uw) is abnormally thickened and contains a high amount of collagen indicated by the light blue stain (right panel). (**F**) A consecutive IHC approach, using an anti-CD31 antibody shows the specific binding to endothelial cells (arrow) and therefore confirms the successful applicability of IHC to PTA diffused murine kidneys.

The excised right lateral fibrotic kidney and the contralateral slightly augmented kidney of a one year old male mouse were stained in PTA solution for 6 days and scanned with the high resolution specimen *Phoenix nanotom* µCT. The data set with a resolution of 12.5 µm displayed the blistered, irregular surface of the affected kidney in comparison to the smooth appearance of the contralateral kidney. Visualization of 3D data sets and planar projections revealed multiple cysts of different sizes within the renal parenchyma without PTA uptake (Fig. 6A–C), whereas cortex, medulla and even renal pelvis showed a diffuse, heterogeneous PTA staining pattern. The associated ureter of the fibrotic kidney was notably thickened and prominent, indicating a high accumulation of PTA. In contrast, the left kidney showed the normal stratification of the organ with decreasing staining intensity from the outer renal cortex to the inner pelvis. The medulla appeared thin and showed large unstained areas, indicating a slow disappearance of the pelvis and medulla with the consequence of functional loss of this kidney (Fig. 6B).

*Masson-Goldner trichrome* (MGT) staining of histological kidney sections confirmed the morphological findings obtained by µCT: characteristic fibrocystic features, a thin parenchyma without distinct differentiation between cortex and medulla and with an irregular central pelvis (Fig. 6D). At higher magnification, multiple cysts of different sizes and stadia were observed, ranging from cystic degenerated glomeruli to cysts with extensive lumina affecting the renal cortex and medulla (Fig. 6E). The wall of the ureter was intensely stained blue, indicating an increased ECM accumulation.

However, by qualitatively comparing the µCT based brightness of the renal cortex of the fibrotic and the unaffected kidney, we found that the darker appearance of the altered fibrotic renal cortex and associated ureter not only indicates PTA staining due to an increase in collagen but also reflects a tubular atrophy and glomerular sparing. Anti-CD31 antibody staining showed a positive and specific labelling of endothelial cells in renal vessels (Fig. 6F), demonstrating that a subsequent IHC approach is possible without changes in pre-treatment or staining protocols after PTA diffusion.

### Density measurements of the cortex reveal and quantify renal fibrosis

**Figure 7:**
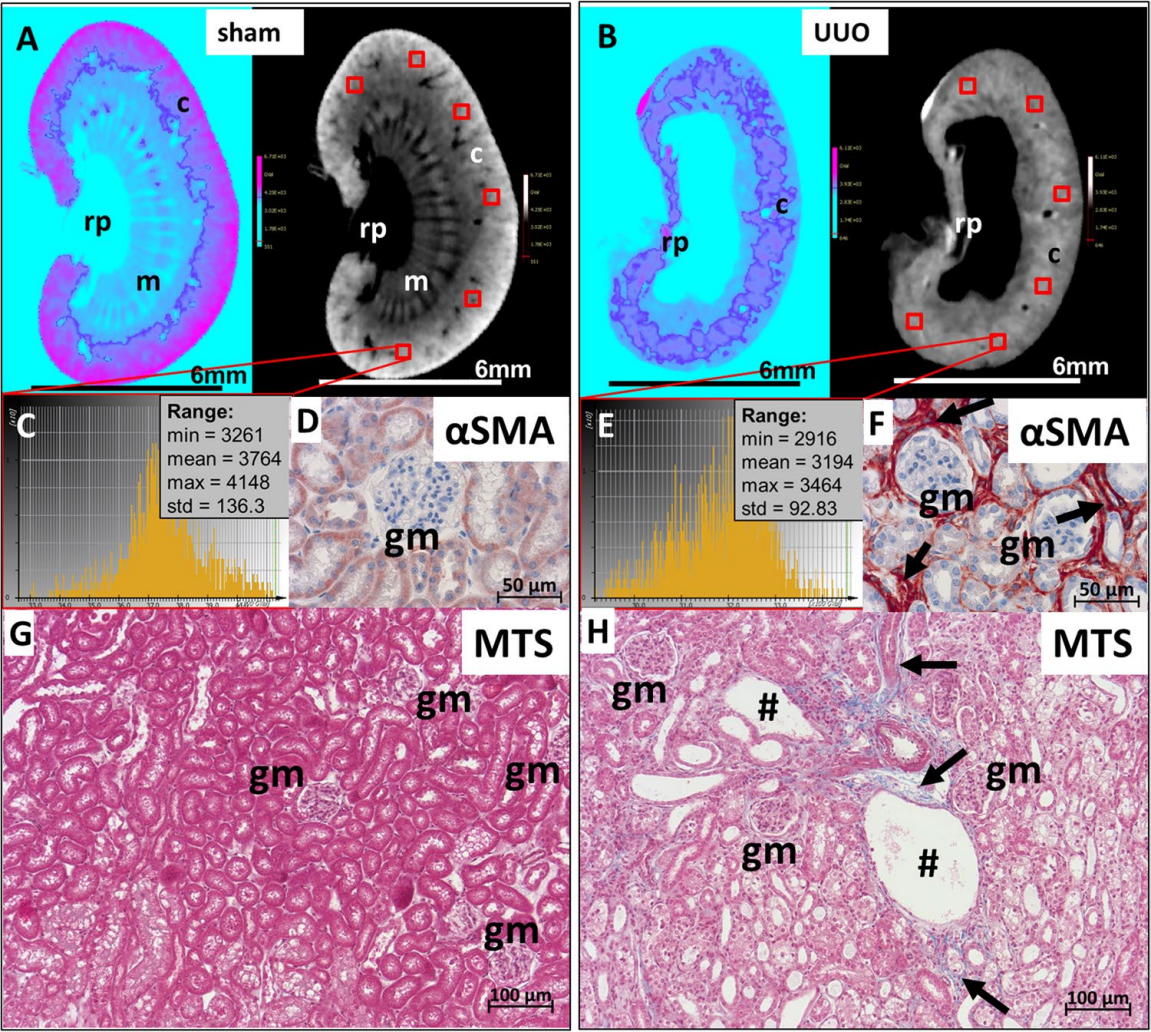
Assessment of renal cortical damage by density analysis based on CT data sets. (**A** and **B**) Two different 2D cross section CT visualization protocols (“*cool*” and “*grey scale*” implemented in *SCRY*) of sham operated versus UUO treated kidneys stained with PTA illustrate the loss of cortical integrity, from the capsule’s site as well as from the inner part of the kidney. The structural loss of renal medulla (m) and renal pelvis (rp) in UUO kidneys is also notable. (**C** and **E**) X-ray opacities in the cortex were confirmed by density histograms, which were generated from six cubes (0.125mm^3^), evenly ditributed in the cortical region. As already observed in the cross sections of the kidneys (**A**,**B**) grey scale values within the renal cortex are lower in UUO kidneys compared to values of sham treated kidneys. (**E**,**F**) Subsequent anti-αSMA immunostaining of these kidney sections confirms the augmented presence of activated fibroblasts (arrows), surrounding tubules and glomeruli (gm) in UUO kidneys as feature of fibrosis (**F**) which were less observed in sham operated kidneys (**D**). **(G**,**H)** To illustrate the structural loss of cortical tissue, micrographs of Masson trichrom staining (MTS) stained kidney sections from sham (**G**) and UUO kidneys (**H**) According to the quantification of the radio-opacity (**A**–**E**), the sham renal cortex appears compactly filled with a dense tubular system and glomeruli (gm). In the UUO operated kidney (**H**) a loose tubular organization with distinct dilated tubules (#), glomerular sparing and overexpression of collagen fibers (arrows), represented by a blue staining within the cortex, is visible. These histopathological findings are in line with the cortical radio-opacity in UUO kidneys and explain the lower grey values in comparison to the unaltered controls.

Next, we explored if the CT based approach allows the determination and quantification of structural alterations within the renal cortex. For this purpose, we analyzed kidneys in a murine model for renal fibrosis by applying unilateral ureteral obstruction (UUO) to the kidney. Through the permanent back pressure of urine into the renal pelvis caused by the ureteral ligation, typical features of renal fibrosis occur with severe atrophy of the renal pelvis and medulla^22^. The respective contralateral kidney (sham) served as an internal control for the UUO undergoing kidneys. Four UUO- and four corresponding sham kidneys excised from four mice were stained with PTA for twelve days and subsequently scanned with the *QuantumFX* CT. The CT data sets showed the UUO typical widening of the renal pelvis with an almost complete extinction of medullary structures. In most specimens, the cortex appeared unaltered, but slightly thinner than in sham kidneys (Fig. 7A,B). Although the renal cortex seemed to be intact, we wanted to quantify the density distribution within the cortex to detect cortical damage, which is not visible at a resolution of 40 µm.

To this end, 3D data sets of virtually cross-sectioned kidneys were laterally orientated and a cube with an edge length of 0.5 mm was set in the area of the renal cortex. 2D views in three different planes confirmed the correct position of the cube at the rim of the renal cortex. A GV histogram was generated from each cube and values such as minimal, mean and maximal GVs were used for further calculation. To assess the ubiquitous GV distribution, GVs obtained from histograms of six different cubes placed within the whole renal cortex were used to determine the mean x-ray opacity for the cortex of each kidney (Fig. 7C,D).

**Figure 8:**
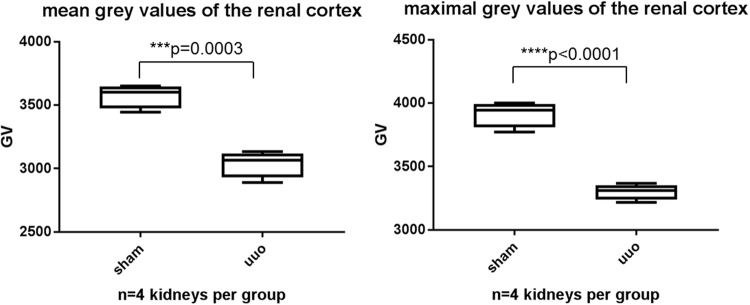
Reduction of radio-opacity in the renal cortex of UUO kidneys in comparison to sham operated kidneys. Box plots of grey values of 6 cortical cubes per kidney are shown (**A**) The mean density value of all analyzed virtual cortical cubes which were generated from the CT datasets is significantly higher in sham treated kidneys (3575 ± 94.5 GV) than in UUO kidneys (3039 ± 105.5 GV). (**B**) The sham operated animals also show the highest values for the maximum radio-opacity (3916 ± 102.1 GV), reflecting a stronger uptake of PTA in the unaffected renal cortex than in the renal cortex of UUO treated mice (3302 ± 61.9 GV).

A significant reduction of density was detectable in all specimens within the renal cortex of UUO kidneys in comparison to corresponding sham kidneys (Fig. 8). The mean GV as a measure for the radio-opacity per 6 cubes of four UUO kidney was 3039 ± 105.5 GV and for the sham kidneys a significantly higher mean value of 3575 ± 94.5 GV was calculated (p = 0.0003). The maximal GV, representing the highest x-ray attenuation within all analyzed voxels, were determined with 3302 ± 61.9 GV for UUO kidneys and 3916 ± 102.1 GV for sham operated kidneys (p < 0.0001).

In order to correlate the results obtained from the isolated cubes with the whole renal cortex volume of each kidney, we determined volume and GV distribution for the whole renal cortex of UUO- and sham treated kidneys. Thus, the cortex volume was obtained by setting the range within the density histogram, which represents the cortex to the maximum grey scale value (Fig. 4B). The mean radio-opacity of the whole cortical volume confirms our results obtained from six isolated cubes: The mean of 3703 ± 133.3 GV for the renal cortex volume of sham kidneys was significantly higher (p < 0.0001) than the GV of UUO kidney renal cortexes (3009.5 ± 80 GV). Analysis of the overall cortex volume revealed a loss of cortical mass of 61.8% ± 22.5% in UUO kidneys compared to sham operated kidneys, assuming that both kidneys of one mouse had the same initial volume. Although the differences of the cortical volumes for UUO and sham kidneys are not significant, the tendency of a decreasing cortical mass is consistent with the observation that renal pathologies and the aging kidney are accompanied with a loss of cortical thickness.

A subsequent Masson Trichrome staining (MTS) and IHC staining with anti-α smooth muscle actin (αSMA)- antibody of paraffin sections of the PTA stained and CT scanned kidneys confirmed the enhanced presence of activated fibroblasts (Fig. 7D,F), and thus increased synthesis of ECM proteins like collagen-1 (Fig. 7G,H) in UUO kidneys. Tubular dilations and an enlarged interstitial space within the renal cortex of UUO kidneys are features of a pronounced fibrosis and confirm the fibrotic remodelling of the renal cortex of UUO kidneys in comparison to the contralateral sham kidneys, which show normal glomerular and tubular morphology, with appropriate epithelial organisation and less activated fibroblasts.

## Discussion

The present study demonstrates the feasibility of cost-efficient PTA based contrast enhancement of isolated murine kidneys for virtual histology by means of high resolution CT. This approach enables the qualitative characterization of healthy kidneys but also malformations and pathological alterations in diseased kidney as well as the quantitative assessment of the volume of each anatomical region. The CT data sets demonstrated a clear morphological stratification of the murine kidney after PTA staining. The unequal contrast in renal capsule, cortex, medulla and pelvis suggests the different grade of tissue compactness. Blood vessels and other structural cavities like the pelvis are not stained by PTA diffusion and therefore show an inherent negative contrast. This even allows the visualization of vessels with a small diameter within the cortex, depending on the resolution of the CT data set. In comparison to standard histological procedures of single slices, which only analyze a small, planar portion of the kidney sections, *PTA contrast enhanced CT analysis* allows the 3D visualization of the data sets in all arbitrary planes and angles, generating a far more complex and complete image of the organ’s anatomy.

By applying intracardial PTA perfusion, we achieved a positive blood vessel contrast, which accentuates PTA filled blood vessels. The hydrous PTA solution was washed out rapidly after isolation of the single organ and a consecutive PTA diffusion staining contrasted the morphological features of the kidney *ex vivo* after complete organ penetration. The subsequent co-registration of the CT data obtained at two time points allowed the depiction of specific vessels after PTA perfusion and the simultaneous representation of the renal morphology, after PTA diffusion. To the best of our knowledge, our approach is the first to provide the possibility of directly examining the perfusion status of the kidney and the associated renal pathological alterations.

A possible way to overcome the time lag between vessel contrast and the morphological contrast is the perfusion of viscous contrast agents like Microfil™. As a lead-containing silicon rubber CT medium, Microfil permanently solidifies within the vessels and thus allows the depiction of the micro architecture of renal blood vessels^23,24^. The similar radio-opaque properties of lead and tungsten are however, a possible drawback of such an experimental set up. An alternative innovative CT based approach using phase contrast has been reported, which enabled the *ex vivo* depiction of human renal carcinoma specimens without contrast agents, making use of the x-ray phase shift that occurs due to different passing characteristics of matter^25^. We showed that the process of PTA staining preserved the tissue without any shrinkage. The observed slight swelling due to the hydrous stain is not kidney specific, as comparable values were obtained in shrinkage studies for PTA stained murine hearts^3^. The mild swelling of the organs is reversed during the chemical dehydration and paraffin embedding processes, which cause shrinkage of the kidneys by almost a fifth of the original volume.

*PTA contrast enhanced CT analysis* does not exclude later conventional histological or IHC procedures. Subsequent paraffinization, sectioning and staining of the PTA perfused kidneys either with H&E and MTS or with antibodies against CD31 and αSMA led to an intensive and specific labeling of the proteins and cells of interest. This can be seen as strong evidence, that PTA itself does not affect the tissue. Histochemical staining of *ex vivo* contrasted tissues was already shown by others^26,27^. Dullin *et al*.^3^ demonstrated IHC staining of PTA stained hearts for the first time. In our study, MGT staining resulted in a ubiquitous light blue staining of the PTA diffused section. As PTA is a component of the standard histological MGT staining, the *ex vivo* PTA staining may lead to a PTA saturation in the sample. In comparison, MTS additionally contains phosphomolybdic acid and resulted in a specific collagen staining, even of delicate fibers. Thus, MTS staining is a better substitute for subsequent collagen depiction after *PTA contrast enhanced CT analysis*.

We show, that *PTA contrast enhanced CT analysis* allows the quantification of i) the volume of each functional region, i.e. renal cortex, medulla and renal pelvis and ii) the thickness of the renal cortex portion. Both could be determined quantitatively based on their different diffusion properties. Histograms of the CT data set offer information about the x-ray opacity and its distribution within the entire organ. Changes in the distribution of the radio-opacity due to an altered accumulation of PTA are measureable (absolutely or relatively) and give evidence on disease specific alterations. Our CT data depicted a reduced cortical thickness and volume in mature mice when compared to adult mice. The reduced cortical thickness has already been described as a surrogate marker of the aging or pathologically altered kidney as observed in CKD, tubulointerstitial disease, microvascular disease or obstructive nephropathy^28,29^ and can be measured in patients by MRI techniques^30^.

We also demonstrated the feasibility of the *PTA contrast enhanced CT histology* to analyze fibrotic murine kidneys. The renal cortex radio-opacity of UUO operated kidneys revealed in comparison to the untreated kidneys of the same animal a notable tissue remodeling, such as the complete loss of pelvic and medullar organization. This is in accordance with the known features of the UUO model, such as a progressive destruction of renal structures and the development of cortical fibrosis^31^. The 3D representation from a fast CT scan revealed an intact and structurally unaffected renal cortex. In contrast, data of the according GV histogram revealed a tremendous decrease of PTA uptake within the cortical rim of UUO kidneys in comparison to the contralateral unaffected kidney, associated with the structural loss within the cortical interstitium. The analysis of the contralateral sham kidney as a control is required in order to define the radio-opacity range of the unaltered renal morphology.

In future, this data based quantification and therefore measurability of renal structural alterations without a clear visual correlate, will allow innovative analytical approaches in experimental studies on nephrotoxicity, transgenic animal models or new therapeutic strategies for the treatment of renal pathologies, which are currently analyzed by planar histological sections^20,32–34^. As we have demonstrated, the PTA staining and subsequent CT scans do not impede a following histological or IHC staining which offers important details on the cellular morphology of diseased tissue. Thus, both approaches can be conducted on the same sample and one does not exclude the other.

Because of technical limitations, currently *PTA contrast enhanced CT histology* does not provide cellular or subcellular resolution. Our approach did not intend to analyze and compare different renal pathologies in detail, but to demonstrate the added value that CT based virtual histology presents above classical histology and IHC, like new information on anatomical features as well as the quantification of volumes of morphological regions, which compensate for the long staining procedure of this method. Because this study was designed as an *ex vivo* end point analysis, time course changes of the 3D structure in the sense of *in vivo* monitoring during disease progression were not realizable.

An *in vivo* use of CT based histology would be very attractive. However, to date PTA has not been used as a contrast agent for *in vivo* imaging. A current drawback of established clinical contrast agents is the fast clearance from the system and thus the limited “staining” of the organs for an equivalently beneficial imaging as our *ex vivo* approach.

However, *PTA enhanced CT analysis* is a valuable prerequisite for further *in vivo* approaches. The definition of certain characteristics of kidney disease such as features of fibrosis, cystic kidneys and loss of cortical thickness by *ex vivo* high resolution CT may lead to the future use of these hallmarks as biomarkers in diagnostic applications and thus facilitate the translation of these into clinic.

Our results show that *PTA enhanced CT* analysis in preclinical studies can help to understand mechanisms of kidney failure by revealing and quantifying pathological alterations in renal diseases and thus support the optimization of therapeutic strategies.

## Materials and Methods

### Animals

*C57BL6/J* mice (male/female; age 4–19.5 months), *C57BL6/N* mice (male; age 2 months) and *FVB/N* mice (female; age 3 months) were obtained from the animal facility of the University Medical Center Göttingen and kept under 12:12 h dark:light cycle with *ad libitum* access to food and water.

To contrast renal vessels, two eight weeks old male *C57BL6/N* mice were euthanized through deep anaesthesia with ketamine (75 mg/kg bodyweight) and xylazine (15 mg/kg bodyweight). After cardiac arrest, mice were transcardially perfused per pump, first with 5 ml PBS (5 ml/min), followed by 40 ml 5% PTA solution (diluted in water; 10 ml/min) and finally with 5 ml 4% paraformaldehyde (PFA diluted in 1xPBS; 5 ml/min).

The *FVB/N* mice in this study underwent surgery for a unilateral ureteral obstruction (UUO) procedure as described earlier by Tampe *et al*. 2015^22^. After 10 days of UUO, the ureteral ligation was removed and the animals recovered for another 7 days before sacrifice and kidney extraction.

All animals, except the perfused ones, were sacrificed using an overdose of carbon dioxide (CO_2_), followed by cervical dislocation.

### Ethics statement

All animal experimental procedures were performed in compliance with the European (2010/63/EU) and German regulations on Animal Welfare and were approved by the administration of Lower Saxony (LAVES) and the Animal Welfare Committee of the University Medical Center Göttingen {Nr. 33.9-42502-04-15/1972, T 10/16}.

### Sample preparation and PTA staining

The excised kidneys were rinsed five times in water and then transferred to 35% ethanol for 1 h, followed by 70% ethanol for 1 h. For staining and fixation, kidneys were placed in staining solution containing 0.7% PTA, 4% PFA and 70% ethanol. Samples were slowly rotated at room temperature (RT). The PTA staining process was checked by CT scans and ranged from four to 18 days to complete penetration of the organs. For storage, samples were transferred to a fresh 70% ethanol solution. For µCT analysis, the PTA stained kidneys were either scanned directly or were scanned embedded in 1% agarose in a 1.8 ml vial. For histology and long term storage, the samples were embedded in paraffin after an automated ascending ethanol series (70%, 90%, xylol, each 1.5 h; dehydration automat, Suesse Labortechnik).

### CT Scanning and Reconstruction

The kidneys (directly or embedded in agarose) were scanned either with the *QuantumFX* *in vivo* microCT (Perkin Elmer, scanning parameters: 90 kV, 200 µA, 40 µm voxel size) or with the Nanotom specimen CT (Phoenix, GE Measurement & Control; scanning parameter: 70 kV, 150 µA, 12.5 µm voxel size). Data reconstruction was performed automatically directly after the scan by the CT-specific software.

### Histology

After CT scanning, the PTA stained kidneys were further processed for histological analysis. Embedded kidneys were first manually removed from the agarose gel. All kidneys were chemically dried using an ascending ethanol series (dehydration automat) and embedded in paraffin. Kidneys were then cut into 2 µm sections and stained as described before, with either haematoxylin/eosin (H&E)^35^, Masson-Goldner trichrome staining (MGT)^4^ or Massons trichrome staining (MTS)^36^.

For immunostaining of alpha Smooth Muscle Actin (α-SMA), deparaffinized and hydrated sections were boiled for 20 min in Target Retrieval Solution (TRS, pH 6.0, Dako), followed by an incubation step of 5 min in ice cold water and two times washing with TBS buffer for 5 min each. Hereafter, sections were incubated 10 min with 3% hydrogen peroxide at RT followed by two washing steps with TBS (5 min each). To prevent unspecific antibody binding a 20 min blocking step with Fish block (SurModics) was applied. The rabbit polyclonal antibody (anti α-SMA, clone 5694, abcam) staining was performed overnight at 4 °C. After washing, the secondary antibody (anti-rabbit-horseradish-peroxidase, undiluted, Histofine) was added for 1 h at RT. Immediately after washing, the enzyme substrate (AEC, BD Pharmingen) was added to the sections for 20 min at RT.

Endothelial staining was performed with a rat monoclonal antibody directed against the endothelial cell marker CD31 (clone SZ31) for 30 min at RT. Subsequently, sections were incubated with an anti-rat-biotinylated secondary antibody (BioLegend) for 1 h at RT, followed by detection with avidin-horseradish-peroxidase (eBioscience), for 1 h at RT.

Irrespective of the antibody staining, sections were counterstained with haematoxylin to color the cell nuclei. Images were obtained with an Axioskop microscope (Zeiss) equipped with a digital camera (Micropublisher 5.0, QImaging Surrey).

### Data analysis

The software SCRY (v5, Kuchel & Sautter GbR) was used for 3D visualization and quantification of the data sets.

### Statistical analysis

Statistical analysis was performed using the unpaired t-Test of the GraphPad Prism software (Version 6). Differences were deemed to be significant when the P-value was <0.05. Analyzed data are represented with standard deviation (±SD).

### Data availability

All relevant data are available from the Open Science framework database, under the doi: 10.17605/OSF.IO/TYXPJ|ARK c7605/osf.io/tyxpj.
